# ESRRA promotes gastric cancer development by regulating the CDC25C/CDK1/CyclinB1 pathway via DSN1

**DOI:** 10.7150/ijbs.57623

**Published:** 2021-05-10

**Authors:** Feng-Nan Li, Qin-Yi Zhang, Ou Li, Shi-Lei Liu, Zi-Yi Yang, Li-Jia Pan, Cheng Zhao, Wei Gong, Yi-Jun Shu, Ping Dong

**Affiliations:** 1 Laboratory of General Surgery and Department of General Surgery, Xinhua Hospital affiliated with Shanghai Jiao Tong University, School of Medicine, No. 1665 Kongjiang Road, Shanghai 200092, China.; 2 Shanghai Key Laboratory of Biliary Tract Disease Research, Shanghai, China, No. 1665 Kongjiang Road, Shanghai 200092, China.

**Keywords:** gastric cancer, oncogene, estrogen-related receptor-α (ESRRA), DSN1 component of MIS12 kinetochore complex (DSN1), cell cycle, CDC25C-CDK1-Cyclin B1

## Abstract

**Background:** Estrogen-related receptor-α (ESRRA) is an orphan nuclear receptor, expressing at high level in exuberant metabolism organs and acting as transcription factor. High expression was found in many malignances but no research was done in gastric cancer (GC), where lipid metabolism disorder is common.

**Methods:** Kaplan-Meier plot was utilized to find the relationship between ESRRA expression and patients' prognoses. The expression level of ESRRA was measured by real-time PCR. The protein expression levels were tested with western-blot and immunohistochemistry. Cell cycle and apoptosis was identified with flow cytometry. RNA-seq, bioinformatics analysis, dual-luciferase assay and ChIP assay were used to predict and validate ESRRA's target gene and binding motif. Animal models were also introduced in our study.

**Results:** ESRRA expression is notably higher in GC cell lines and high ESRRA levels are correlated to poor prognoses. ESRRA silencing decreased GC cell viability, migration, and invasion capacities. Its downstream gene DSN1 was spotted by RNA-seq and confirmed by later bioinformatics analyses, dual-luciferase, and ChIP assays. Western-blot showed G2M arrest caused by ESRRA silencing was via CDC25C-CDK1-Cyclin B1 pathway.

**Conclusion:** ESRRA/DSN1/CDC25C-CDK1-Cyclin B1 is of great importance in GC development. ESRRA could be a potential target as well as prognostic marker in GC.

## Introduction

Gastric cancer (GC) ranks fifth place in the most frequently diagnosed cancer and is the third leading cause of cancer death globally in 2018, which caused around 783,000 deaths in 2018. GC's age-standardized incidence rate is extremely high in Eastern Asia, among which Republic of Korea's rates rank first worldwide in both sexes 1. Even though diagnosis and treatment methods have developed a lot, the prognosis of GC is still dismal due to its unapparent early clinical manifestations. In China, merely 20% GC patients are diagnosed in early stages, and the overall 5-year survival rate is less than half 2. Consequently, it is crucial to explore the underlying mechanism in GC development as to provide ideas in target treatment and eventually improve GC prognosis.

It has been reported that when focusing on mortality and survival periods differences in various races, African American (AA) cancer patients' prognoses are worse than European American. Arun Sreekumar et al. performed Gene Set Enrichment Analysis (GSEA) coupled to transcription factor enrichment to identify a common biological driver of pan-cancer with The Cancer Genome Atlas data set. GSEA revealed oxidative phosphorylation (OXPHOS), DNA repair, and G2M checkpoint are enriched exclusively in AA cancer patients, and highly positive enrichment was shown in gastric cancer. Besides, the transcriptional factor estrogen-related receptor alpha was identified as the most significant and common enriched transcription factor in pan-cancer screen including gastric cancer 3. For this reason, we determined to explore the role that estrogen related receptor alpha play in GC.

Estrogen related receptor alpha, also known as ESRRA, ERR1, ERRa, ERRalpha, ESRL1 and NR3B1, is an orphan nuclear receptor. Even it was found closely related to Estrogen receptor alpha, it doesn't interact with natural estrogens, estrogens' derivatives or any other steroid hormone 4. However, there are evidences that ESRRA is a transcription factor, which interact with estrogen responsive elements (EREs) and estrogen-related responsive elements (ERREs) thus controlling vast genes related to lipid and glucose metabolism, as well as mitochondrial biogenesis 5. Intriguingly, evidences witness ESRRA contributes to tumorigenesis. It has been reported that ESRRA over-expression is related to poor prognosis in various cancers, such as breast cancer 6, glioma 7 and gallbladder cancer 8, via accelerating cancer cell growth and enhancing migration and invasion capacity. However, few researches have explored the role of ESRRA plays in gastric cancer.

In our study, worse clinic prognosis in relation to higher ESRRA expression was identified. We also observed that ESRRA depletion in GC cell lines impaired cell viability, invasion, and migration capacities. Meanwhile, cell cycle arrest and elevated apoptosis rate were also presented when ESRRA was knockdown. As to identify downstream effectors, we perform RNA sequencing (RNA-seq) with using ESRRA knockdown HGC 27 and MGC 803 human gastric cancer cell lines and untreated HGC 27 and MGC 803 cell lines. By analyzing the result, we found that DSN1's fold change was in accordance with ESRRA's expression level.

Considering DSN1 expression level was downregulated when ESRRA was knocked down, we wondered if ESRRA is DSN1's transcription factor. After the hypothesis built, we explored Harmonizome, a collection of processed datasets gathered to serve and mine knowledge about genes and proteins from over 70 major online resources, to find if there is any EREs or ERREs motifs in DSN1 promoter region for ESRRA to bind. Since it was established that estrogen receptor and estrogen related receptor beta can regulate DSN1 by binding to its promoter region, we predicted the possible binds sites for ESRRA within the promoter of DSN1 with USCS Genome Browser (http://genome.ucsc.edu/) as well as JASPAR (http://jaspar.genereg.net) and conducted ChIP analyses, q-PCR and nucleic acid electrophoresis to validate the results.

Afterwards, we performed rescue assays by overexpressing DSN1 in ESRRA knocking down GC cell lines *in vitro* and *in vivo*. Cycle arrest and molecule markers related to cycle arrest were reversed to a degree but apoptosis status was not restored.

## Materials and methods

### Patients and clinicopathologic data

Gastric cancer tissue and adjacent cancer mucosa were obtained from the Department of General Surgery in Xinhua Hospital affiliated with Shanghai Jiao Tong University School of Medicine (Shanghai, China). No patient underwent chemotherapy, radiotherapy, or immunotherapy before surgery. All experiments were approved by the Research Ethics Committee of the Xinhua Hospital affiliated with Shanghai Jiao Tong University School of Medicine (Approval No. XHEC-F-2020-024) and written informed consents were obtained from all patients who contributed samples to this study.

### Cell culture and chemicals

Human gastric cancer cell lines (HGC27, BGC823 MGC803 and SGC7901) and normal gastric epithelial cell line GES-1 were acquired from the Shanghai Key Laboratory of Biliary Tract Disease Research, Shanghai, China. HGC27, BGC823, MGC803, SGC 7901, and GES-1 were cultured in RPMI 1640 (Hyclone) with 10% fetal bovine serum (FBS) (Invitrogen Gibco) at 37 °C in humidified incubator containing 5% CO_2_.

### Quantitative real-time PCR

Total RNAs were extracted from tissue samples, HGC27, BGC823, MGC803 and GES-1 cells by using Trizol reagent (Invitrogen). cDNA was synthesized by PrimeScript Reverse Transcriptase (Takara) according to manual instruction. The primers used for the detection of ESRRA, DSN1 and GAPDH (internal control) expression were: ESRRA forward primer, 5'-GAGATCACCAAGCGGAGACG-3'; reverse primer, 5'-ATGAGACACCAGTGCATTCAC-3'. DSN1 forward primer, 5'-CTCAGCCGGTCTATCAGTGTC-3'; reverse primer, 5'-AGTGTCCCTTAGGAAAGGTTCAA-3'; GAPDH forward primer, 5'-GGAGCGAGATCCCTCCAAAA-3'; reverse primer, 5'-GGCTGTTGTCATACTTCTCATGG-3'. Target gene expression level was detected using SYBR Green method and the StepOnePlus Real-time PCR System (Applied Biosystems).

### siRNA, shRNA and plasmid construction

3 pairs siRNA were designed and efficiency were tested with Quantitative real-time PCR. The sequences are 5'-GCGAGAGGAGUAUGUUCUA-3', 5'-GAGAGGAGUAUGUUCUACUAA-3' and 5' GAGAGAUUGUGGUCACCAU-3'. ESRRA was silenced in HGC 27 and MGC 803 with Rfect (BAIDAI) according to instructions. The most efficient pair was chosen to create the ESRRA short hairpin RNA (shRNA)-silencing cell lines. The following shRNA sequence was designed against the ESRRA gene: 5'- GAGAGAUUGUGGUCACCAU -3' and negative control was 5'-TTCTCCGAACGTGTCACGT-3'. These specific shRNA sequences were packaged into PGMLV-SC5 lentivirus constructed by Genomeditech then transfected into HGC27 and MGC803 according to instruction provided by Genomeditech. The transfection efficiency in the infected cells was validated by qRT-PCR. The plasmid pDSN1 and pESRRA, which encode the full-length cDNA of the DSN1[NM_001145316.1] and ESRRA [NM_004451.5] were constructed by Genomeditech. The empty vector (pCMV6) was used as negative control. Lipofectamine 3000 transfection reagent was used for plasmid transfection according to the manual (Invitrogen).

### Cell viability assay

CCK8 assay kit (Yeasen, Shanghai) was used to measure cell proliferation potential. Gastric cancer cells were seeded in 96-well plates at the density of 1000 cells per well with 100 μL RPMI 1640 medium with 10% FBS. At 6, 24, 48, 72, 96 and 120 hours, complete medium were discarded and we incubated each well with 10 μL CCK-8 reagent for 2 hours at 37 °C in the dark. Afterwards, the absorbance value (OD) was measured at 450 nm with SpectraMax 190 Microplate Reader. EdU staining was performed using an EdU kit (BeyoClick™ EdU Cell Proliferation Kit with Alexa Fluor 488, Beyotime, China), HGC 27 and MGC 803 were seeded in 24-well plate at density of 2 × 10^4^ cells/well and cultured for 24 h. Afterwards, cells were incubated with EdU, paraformaldehyde 0.3% Triton X-100 in succession according to instruction provided by Beyotime. Subsequently, the cells were incubated with the Click Reaction Mixture for 30 min at room temperature in a dark place and then incubated with Hoechst 33342 for 10 min. Pictures were captured by Olympus fluorescence microscope system.

### Colony-forming assay

Gastric cancer cells were cultured in 6-well plates at the density of 500 cells per well with 2 mL complete medium. After 2 weeks, proliferating colonies were washed 2 times softly with PBS and fixed with paraformaldehyde for 15 minutes. Then paraformaldehyde was washed and the colonies were stain with crystal violet for another 15 minutes. Only more than 50 cells' colonies were counted.

### Cell invasion and migration assays

Cell migration and invasion potencies were studied by wound-healing and Transwell. In wound-healing assay, gastric cancer cells were seeded into 6-well plates and grow reach to 100% confluence, the single layer cells were scratched straightly with pipette tip. At 0 hour and 24 hours the distance covered by migrated cells was identified. 24-well Transwell chamber inserts (Corning, Corning, NY, USA) and Corning BioCoat Growth Factor Reduced Matrigel Invasion Chambers (Corning) were used respectively in migration and invasion assays. 6 × 10^4^ HGC 27 and 4 × 10^4^ MGC 803 were resuspended in 200 μL RPMI 1640 medium and laid into upper chamber, meanwhile 750 μL RPMI 1640 with 10% FBS was added into lower chamber. The plate was incubated for 24 hours at 37 °C in humidified incubator containing 5% CO_2_. Afterwards, medium was discarded, cells were fixed with paraformaldehyde for 15 minutes and then stained with crystal violet for 15 minutes. Random fields were captured under microscope at 100×.

### Apoptosis and cell cycle assay

We used flow cytometry to perform apoptosis and cell cycle assays. In apoptosis assay, samples were analyzed by using Annexin V-FITC kit according to the manufacturer's protocol (BD Biosciences, CatLog: 556547). After incubation for 30 minutes in the dark at room temperature, samples were analyzed by flow cytometry (CytoFLEX, Beckman Coulter). In cell cycle assay, we use Cell Cycle Analysis Kit (Beyotime) to analyze the cell cycle distribution. Briefly, gastric cancer cells were harvested and washed twice with cold PBS, then fixed with 70% ethanol at 4 °C overnight. Afterwards, samples were stained with PI and RNase A solution for 30 minutes at 37 °C in the dark. In the end, the cell cycle distributions of these samples were detected by flow cytometry (CytoFLEX, Beckman Coulter).

### Western-blot analysis

Protein was isolated with RIPA buffer with PI cocktail (Beyotime) and quantification was done using BCA assays (Beyotime). The supernatant was separated by SDS-PAGE and transferred onto PVDF membranes (Millipore). Primary antibodies were obtained from Abclonal, Cell Signaling and Invitrogen and all primary antibodies were rabbit. After the membranes were blocked with 5% skim milk in TBS-T at room temperature for 1 hour and washed 5 minutes for 3 times, they were incubated with primary antibodies overnight at 4 °C. The next day, we discarded primary antibody and washed membranes with TBS-T for 10 minutes 3 times and then incubated them with goat anti-rabbit HRP-conjugated secondary antibody for 1 hour at room temperature. Afterwards, membranes were washed another 10 minutes 3 times with TBS-T and measured using Gel Doc 2000 (Bio-Rad).

### mRNA Library Construction, RNA-seq and data analysis

The NC and siESRRA group for both HGC27 and MGC803 cells' total RNA extraction were performed using Trizol reagent (Invitrogen) then qualified and quantified by Nano Drop 2000 (Thermo Fisher Scientific, MA, USA). Oligo(dT)-attached magnetic beads were used to purified mRNA, and cDNA was generated using random hexamer-primed reverse transcription and amplified by PCR. The products were purified by Ampure XP beads and validated on the Agilent Technologies 2100 bioanalyser for quality control. The double stranded PCR products from previous step were heated denatured and circularized by the splint oligo sequence into single strand circle DNA (ssCir DNA) formatted as the final library. Construction of RNA-seq library and RNA-seq was completed by BGI company (Shenzhen, China). The differentially expressed genes were identified under absolute log2 (FC) values > 0.5, p < 0.05 were considered as significant.

### Chromatin immunoprecipitation

From RNA-seq results, DSN1 expression fold change was identified as statistically significant. Considering ESRRA is a transcription factor, we used JASPER to predict the motif and the most possible binding sites for ESRRA in the promoter of DSN1. The promotor sequences were obtained from UCSC Genome Browser Gateway and primers for the promising binding sites were designed with the help of Primer-Blast (https://www.ncbi.nlm.nih.gov/tools/primer-blast/). Afterwards, ChIP Assay Kit (Beyotime) was used in HGC27 cell line validate the relationship between ESRRA protein and DSN1. DNA electrophoresis and ChIP-qPCR were used to detect DSN1 promoter sequence enrichment pulled by ESRRA protein antibody.

### Dual-Luciferase assay

Dual-Luciferase assay was conducted to directly analyze the transcriptional regulation of DNS1 by ESRRA. DSN1 promoter containing wild type and mutant were cloned in pGL3 vectors separately by Genomeditech and the sequences are introduced in supplementary materials. Cells were cultured for 24 h and luciferase activity was measured with Dual-Luciferase Reporter Assay System (Promega). Results were normalized by renilla luciferase activity.

### Subcutaneous xenograft model in nude mice

After approved by the Ethics Committee of Xinhua Hospital Affiliated to Shanghai Jiao Tong University School of Medicine, Male nude mice (4 weeks old) were acquired from Shanghai Laboratory Animal Center of the Chinese Academy of Sciences (Shanghai, China) and housed at pathogen-free condition. Three groups each containing 5 mice were injected 5 × 10^6^ MGC 803 cells, Lv-shESRRA MGC 803 cells and Lv-shESRRA+pDSN1 (Lv-shESRRA+DSN1 oe) MGC 803 cells. 4 weeks after injection, mice were killed and subcutaneous tumors were dissected for size measuring, weighing and IHC assays.

### Statistical analysis

Statistical analyses were performed with Prism 8 (GraphPad Software) and SPSS 24 (SPSS Inc.). Student's t test was performed between two groups and analysis between multiple groups was conducted by one-way analysis of variance, data are recorded int the form of mean ± SD. P values of <0.05 were considered statistically significant. We also use χ^2^ test to detect the correlation between ESRRA expression and clinicopathologic data. Also, Kaplan-Meier test for the univariate survival analysis.

## Results

### ESRRA is overexpressed in GC specimens and cell lines

First, we explored the ESRRA expression status in 27 different human tissues and found stomach's ESRRA expression level ranks 6th among them (Figure S2D). Later, we examined its differential expression between gastric cancer and normal tissues with GEPIA2 (http://gepia2.cancer-pku.cn/), which is based on The Cancer Genome Atlas (TGCA) and confirmed ESRRA is overexpressed in gastric cancer comparing with normal tissues (Figure S2E). Finally, we explored the expression differences among various solid tumors and found that ESRRA expression level in gastric cancer is generally higher than colorectal adenocarcinoma, breast cancer and lung cancer according to Jaiswal Multi-cancer Statistics (Figure S2F).

**Figure 1:**
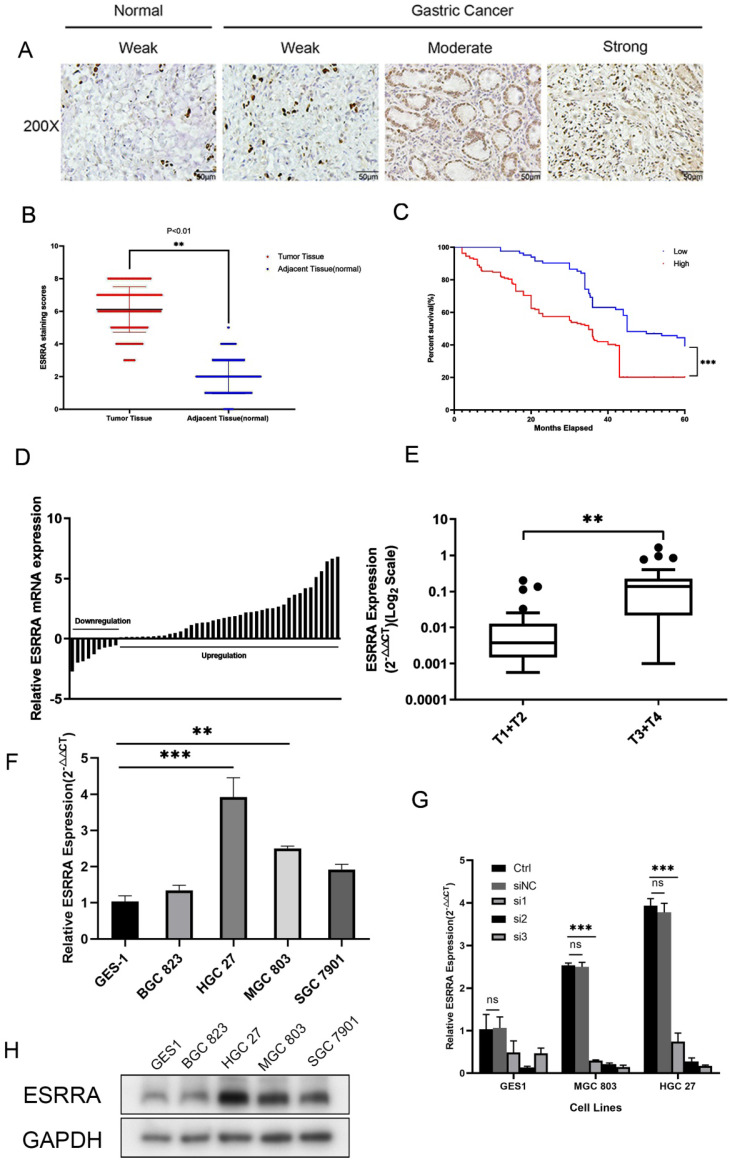
** ESRRA is upregulated in GC and related to poor prognosis.** A. Immunohistochemical staining for ESRRA protein expression. Normal epithelial mucosa and GC staining weak, moderate, and strong were scored with 0, 2, 4 and 6.B. Scatterplots of the total score in GC and adjacent tissues. Total score was calculated as the sum of the intensity parameters. The expression of ESRRA in GC tissues was significantly higher than that in adjacent tissues (P<0.001). C. Overall survival (OS) of GC patients classed by ESRRA expression intensity. Moderate and strong staining were reckoned as positive and weak staining was considered as negative. Patients with positive staining showed poorer OS. D. Expression of ESRRA in 50 pairs of GC and their adjacent mucosa. The expression levels of ESRRA were measured with qRT-PCR. E. Box plot of ESRRA expression with T1+T2 grade and T3+T4 grade. F. Expression levels of ESRRA in GC cell and normal gastric mucosa cell lines (BGC 823, HGC 27, MGC 803, SGC 7901 and GES1). G. Transfection efficiency of 3 siRNA for ESRRA knock-down was determined via qRT-PCR and si3 showed greatest capacity. H. Relative protein expression levels of ESRRA in GC cell lines. All the experiments were repeated 3 times.

In order to explore the expression status in clinical specimens, we performed qRT-PCR in 50 pairs randomly selected human GC tissues and adjacent normal tissues and calculated GC tissue/adjacent normal tissue relative ESRRA mRNA expression. Evidence showed ESRRA was upregulate in a large scale in tumor tissues (Figure 1D). In addition, ESRRA expression level in gastric cancer with grade T3 and T4 was significantly higher than T1 and T2 (Figure 1E). As we have already known that ESRRA is overexpressed in clinical specimens, we detected the expression level of ESRRA in 4 GC cell lines together with GES 1, normal gastric epithelial cell line via qRT-PCR and western-blot. Compared to GES-1, ESRRA was highly expressed in GC cell lines in to different degrees (Figure 1F, 1H). HGC 27 and MGC 803 were chosen to this experiment for their higher ESRRA expression and we knocked down ESRRA with 3 sequences of siRNA and performed qRT-PCR only to found si3 showed greatest knock-down capacity (Figure 1G). To avoid off-target effect, we chose si2 and si3 sequences, which are named as si-ESRRA1 and si-ESRRA2, to conduct western-blot assays (Figure S1E). The sequence of si3 was chosen to construct Lv-shESRRA and we also validated the efficiency of Lv-shESRRA by qRT-PCR (Figure S1A, S1B).

### High ESRRA expression relates to poorer prognosis in GC patients

We detected expression level of ESRRA in GC and adjacent mucosa cell nuclear with IHC (Figure 1A, B). Among 246 patients, 163 showed high level expression. We also found that tumors' ESRRA expression levels were higher than adjacent tissues. Higher ESRRA expression level was related to tumor invasion depth, lymph node metastasis, distant metastasis as well as TNM stage statistic significantly (Table 1). To explore the relationship between ESRRA expression and prognosis of GC, firstly we utilized Kaplan-Meier plotter (http://kmplot.com/) to detect whether ESRRA mRNA expression level matters prognosis. Result produced by Kaplan-Meier plotter disclosed that higher expression was significantly associated with a shorter OS for GC patients (hazard ratio=1.23; 95% confidence interval, 1.02-1.48; P=0.034) (Figure S2A) as well as first progression (PF) and post progression survival (PPS) (Figure S2B, S2C). Then we plotted survival curve with our clinicopathologic data, same conclusion was drawn that higher ESRRA expression is related to dismal prognosis (Figure 1C).

### Knocking down ESRRA in GC cell lines attenuates viability and proliferation ability

**Figure 2:**
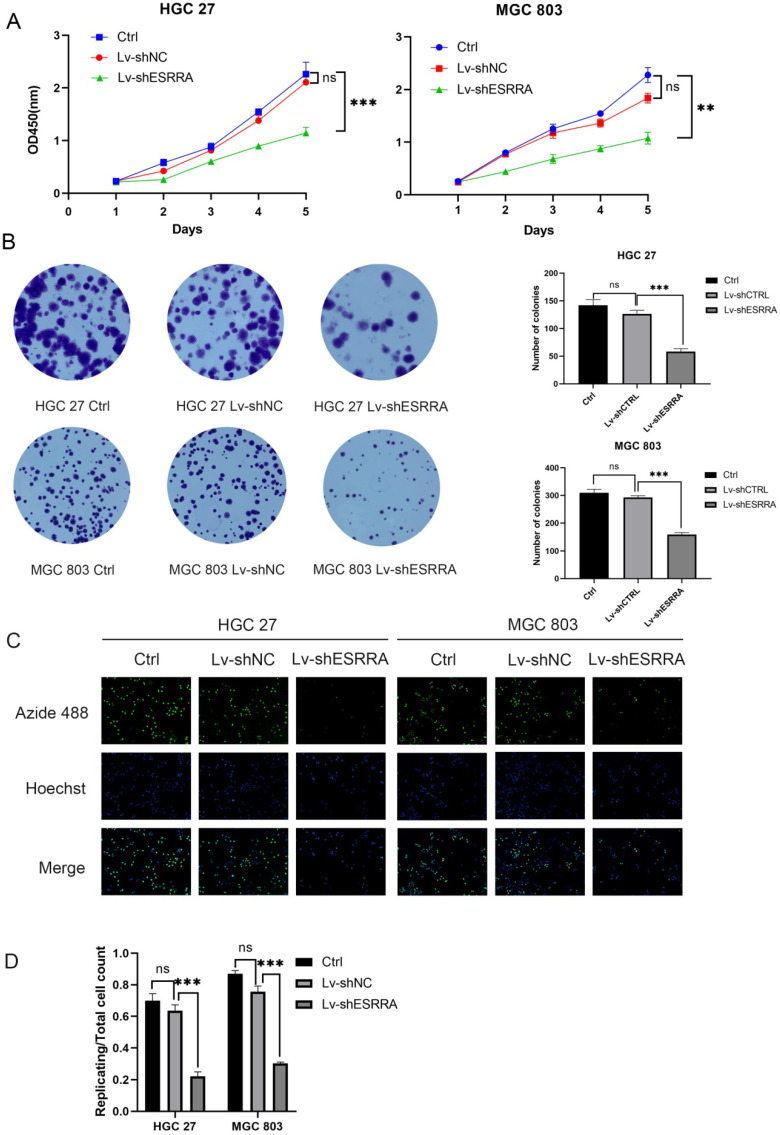
** ESRRA depletion had negative effects on the viability, colony formation, proliferation of GC cells.** A. Compared with controls and negative controls, the viability measured with CCK8 of HGC 27 and MGC 803 cells transfected with Lv-shESRRA was significantly inhibited. B. Silencing ESRRA significantly decreased the colony formation ability of HGC 27 and MGC 803 cells. The colony formation number of HGC 27 and MGC 803 cells were counted and compared in the diagrams. C, D. EdU assays showed that ESRRA silencing group had worse proliferation ability. The replicating DNA were stained with Azide 488 and nuclei were counter stained with DAPI (Images were captured at 20X). Significant differences are indicated by*P<0.05, **P<0.01, and ***P<0.001. All the experiments were repeated 3 times.

CCK-8 and colony formation assays were conducted to measure if GC's viability were impaired when ESRRA was knocked down. Firstly, MGC 803 and HGC 27 were chosen in our experiment for their higher ESRRA expression levels. Secondly, we constructed ESRRA-knockdown cells with 3 ESRRA-specific si-RNAs meanwhile determining the most efficient sequence among them. NC si-RNA were also applied to avoid off-target effect. The CCK-8 assays revealed that GC cell viabilities were inhibited in knocking-down cell lines to a significant degree and we found that No.3 ESRRA-specific siRNA showed best potency (Figure S1C, S1D), which also showed greatest ability in knocking down ESRRA. CCK-8 assays were repeated with Lv-shESRRA, who shared the same sequence with si3 and colony formation assay were also performed (Figure 2A, 2B), we observed the relationship between ESRRA downregulation and decreased viability as well as proliferation ability. EdU results showed the tendency that ESRRA silencing weakened proliferation ability of GC cell lines (Figure 2C). We also overexpressed ESRRA in MGC 803 and HGC 27, the upregulated groups in both these two cell lines showed vigorous viability (Figure S1F, S1G).

### ESRRA promotes GC cell migration and invasion by inducing epithelial mesenchymal transition (EMT)

**Figure 3:**
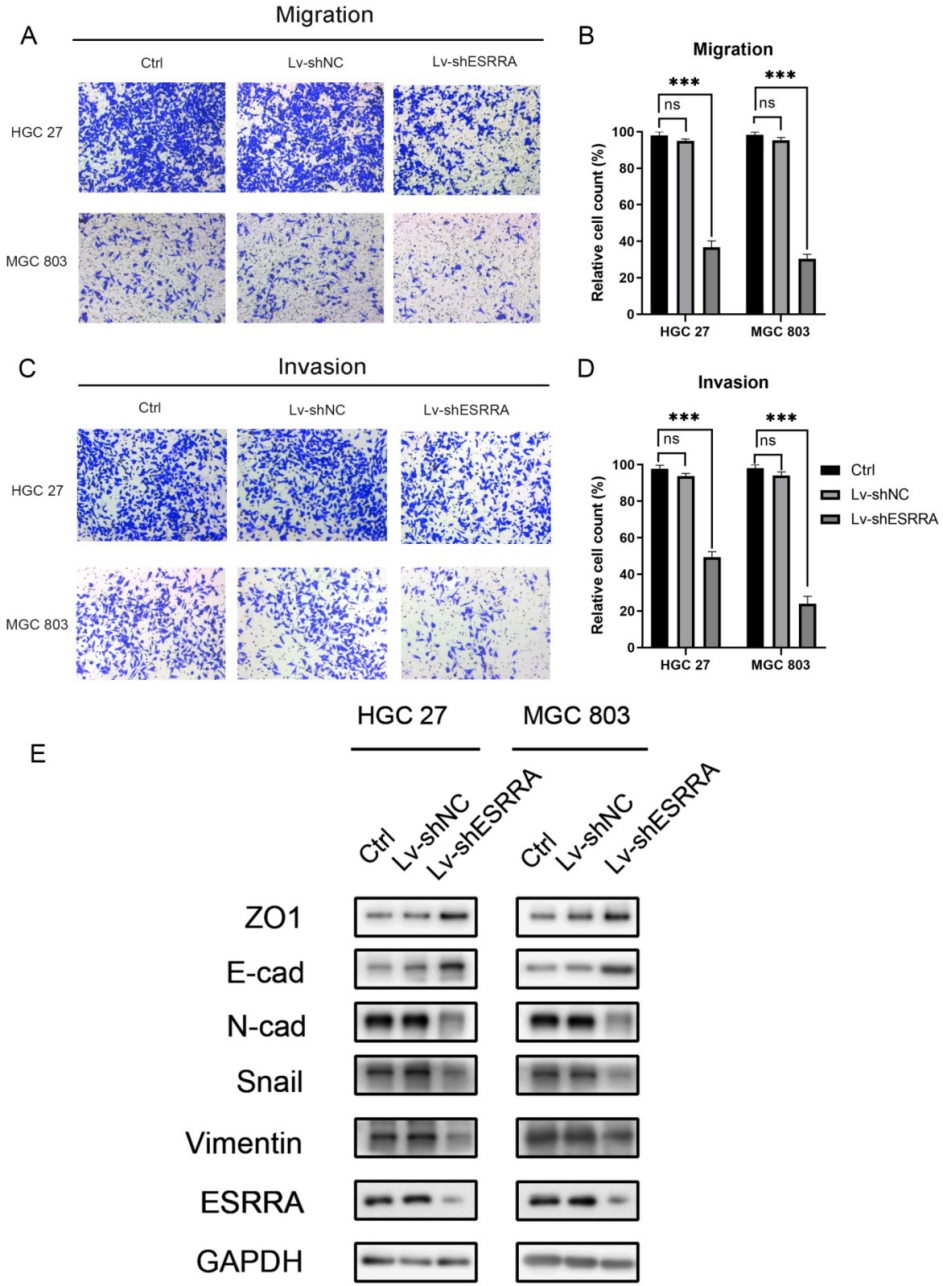
** ESRRA depletion inhibited GC cell migration and invasion.** A, C. Transwell assays, with and without Matrigels, were conducted to explore the effects of ESRRA downregulating on migration and invasion capacities of HGC 27 and MGC 803 cells. B, D. The number of cells passed through the chamber membranes was counted and compared in the diagrams (*P<0.05, **P<0.01, and ***P<0.001). E. Protein expression levels of ZO1, E-cadherin, N-cadherin, Snail, Vimentin were examined by western-blot. All the experiments were repeated 3 times.

Transwell migration and Matrigel cell invasion assays were conducted to figure out whether ESRRA expression level impacts on cancer cell metastasis capabilities. As shown in Figure 3A-D, HGC 27 and MGC803 transfected with Lv-shESRRA showed significantly weakened migrative and invasive abilities. In addition, upregulating ESRRA favored GC's migrative and invasive capacities (Figure S1H, S1I, S1J, S1K). Since we observed this outcome, we conducted western-blot to detect EMT markers in ESRRA downregulated HGC 27 and MGC 803. The expression level of snail, an oncogene which is also a promoter of EMT by repress E-cadherin directly 9, was measured in our study. Considering recent studies have discovered changes in the level of various cadherin, which also known as cadherin switches, can be used to monitor EMT. Especially, E-cadherin to N-cadherin switch in cancer cells relates to cancer progression 10. We did further research in E-cadherin and N-cadherin expression level change. Besides, it has been documented that loss function of epithelial tight junction such as ZO-1 and acquisition of mesenchymal vimentin also favors migration and invasion 11. We also focused on their changes. Results revealed that epithelial marker E-cadherin was highly expressed in ESRRA depleted cell lines and Vimentin, Snail and N-cadherin were lower in Lv-shESRRA transfected lines. Besides, ZO-1 was also seen downregulated in ESRRA silencing groups (Figure 3E). In conclusion, ESRRA promotes GC cell migration and invasion via EMT.

### ESRRA promotes GC development by regulating DSN1

**Figure 4:**
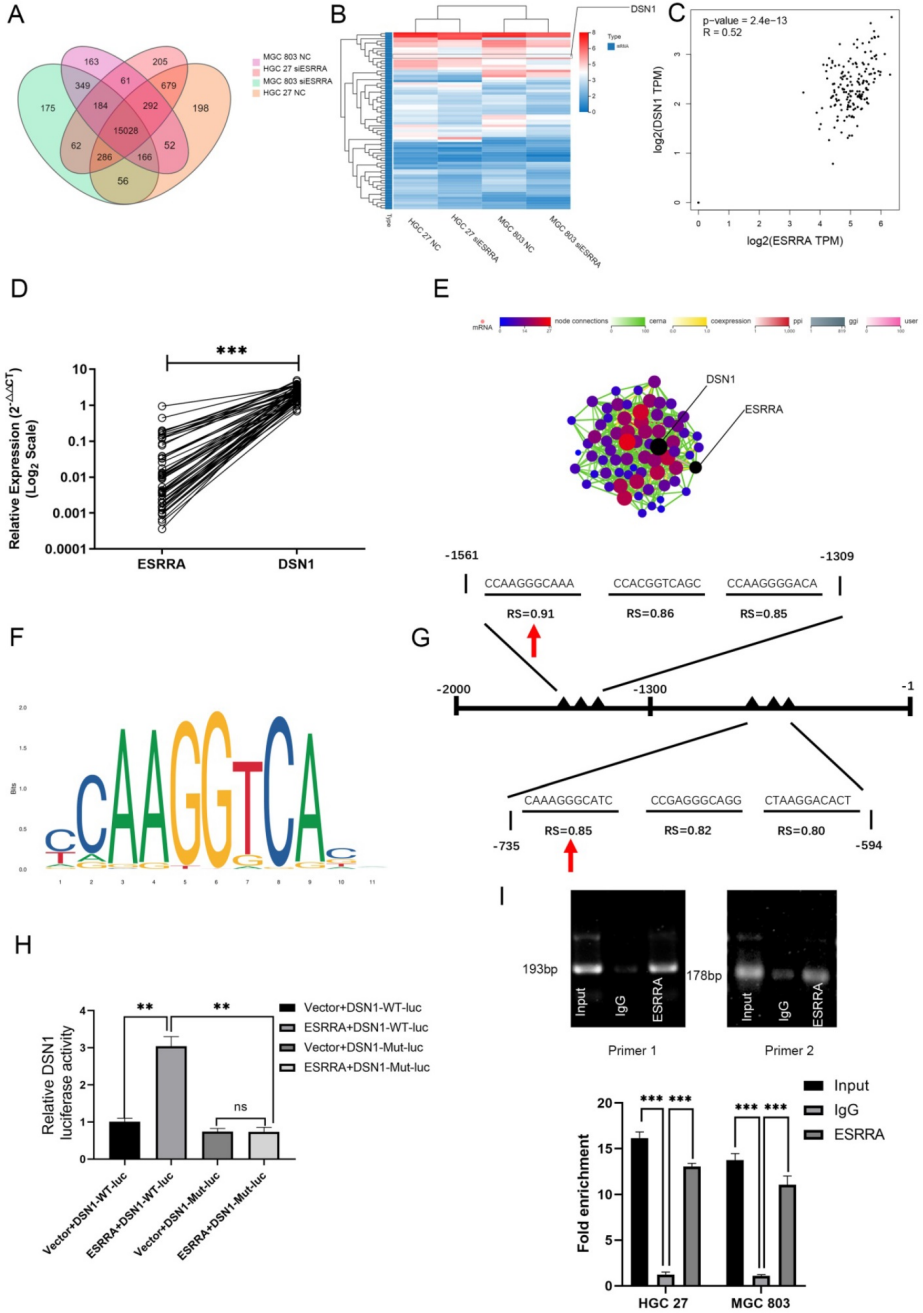
** ESRRA promotes GC development by regulating DSN1.** A. Venn diagram representing RNA-seq results. Four group were established according to whether treated by siESRRA or not and they are HGC 27 NC, HGC 27 siESRRA, MGC 803 NC and MGC 803 siESRRA. 15028 genes were spotted among them. B. FPKM≥1, log2 |siESRRA/NC| foldchange≥0.5 and Q≤0.05 as inclusive criteria for the 15028 genes which are possibly affected by ESRRA silencing and 71 genes were screened out. Heatmap was drawn and DSN1 was reckoned as the most possible gene regulated by ESRRA for its apparent change fold. C. Correlation Analysis between ESRRA and DSN1 was compute via Gepia2 with GTEx database. (Pearson coefficient was used in this figure and Spearman coefficient was used R=0.41, P=1.4e-08). And when ESRRA and DSN1 were normalized by GAPDH, R=0.96, P=0 (not shown in figures). D. The correlation of mRNA levels between ESRRA and DSN1 in 50 randomly chosen specimen were measured and strong correlation between ESRRA and DSN1 was spotted. E. Network interaction analysis showed that DSN1 could also be seen as the hub gene. F, G. 2 potential binding motifs for ESRRA in DSN1's promoter regions were predicted with the help of JASPAR and the accurate sites were also spotted. H. Activity of WT-DSN1 promoter luciferase reporter was significantly enhanced by ESRRA and mutant DSN1 promoter luciferase reporter was unaffected by ESRRA. All the experiments were repeated 3 times. I. Primer 1 and 2 were designed to amplified CCAAGGGCAAA and CAAAGGGCATC motifs. Results of ChIP-DNA electrophoresis and ChIP q-PCR confirmed that ESRRA binds to both motifs. Input refers to amplified DSN1 from total chromatin; IgG, chromatin fragments pulled down by anti-IgG antibody, which acted as negative control. All data are presented as mean ± SD and all the experiments were repeated 3 times. RS, relative score. *P<0.05, **P<0.01, and ***P<0.001.

In order to elucidate the how ESRRA affect GC development, we started analysis with gene expression profiling by RNA sequencing (RNA-Seq) to evaluate its effect on the transcriptome. We set HGC 27 NC, HGC 27 siESRRA, MGC 803 NC and MGC 803 siESRRA with two replicates of each group. As shown in Figure 4A, 15028 genes differentially expressed in all these four groups. We set FPKM≥1, log2 |siESRRA/NC| foldchange≥0.5 and Q≤0.05 as inclusive criteria for searching genes which are possibly regulated by transcription factor ESRRA. Heatmap is presented in Figure 4B and Network interaction including PPI (min score 500), Target (min score 2), Coexpression (min score 0.8), ceRNA (min score 10), GGI (min score 1) and RNAplex (max MFE -100) analysis are also conducted. From the analysis above we noticed that the the FPKM and foldchange of gene DSN1 component of MIS12 kinetochore complex (DSN1) affected most when ESRRA was downregulated. In HGC27NC-vs-HGC27ESRRAsi group, log_2_Ratio is -0.623926508997168 and Q value is 8.26617000720535e-26; In MGC803NC-vs-MGC803ESRRAsi group, log_2_Ratio is -0.791597228394029 and Q value is 4.04791606755435e-43. In Network interaction analysis, DSN1 could also be seen as the hub gene (Figure 4E). We also computed the correlation of ESRRA and DSN1 in GTEx database via GEPIA 2. As shown in Figure 4C, significant positive correlation was found. We also performed qRT-PCR with 50 GC specimens mentioned above, which were collected by our center to validate the relationship between ESRRA and DSN1 in real world. Positive correlation with statistically significant was observed (Figure 4D). Based on all these results, we reckoned that DSN1 is regulated by ESRRA and then confirmed the results by western-blot. Considering ESRRA is a transcription factor, which may regulate expression of DSN1 by binding potential EREs or ERREs in its promoter. We searched transcription factor information of DSN1 by using Harmonizome (http://amp.pharm.mssm.edu/Harmonizome/) and found that ESRRB and ESR1 were predicted as transcription factor of DSN1 in CHEA Transcription Factor Targets dataset and ENCODE Transcription Factor Binding Site Profiles dataset respectively. Evidences above validated there were EREs and ERREs in DSN1's promoter and we predicted 2 potential binding motifs for ESRRA within the sequence using JASPAR (http://jaspar.genereg.net/), including “CCAAGGGCAAA” and “CAAAGGGCATC” (Figure 4F). The relative scores for these 2 motifs were 0.91 and 0.85 (Figure 4G). Furthermore, dual-luciferase assay showed that ESRRA transactivation of the DSN1 transcription (Figure 4H). In addition, we designed 2 pairs of primer sequences: site 1 forward, 5'- AGTTTAAGAGGCAGGCAAGCA-3'; site 1 reverse, 5'- GCAGTTGTGGTCCTTGGAAAA-3'; site 2 forward, 5'- TTGGACTGTGGGCTGCTTAT-3'; site 2 reverse, 5'- CCCAGGAGCCCAATCTGTTC-3' for later ChIP validation. Results for ChIP-DNA electrophoresis and ChIP-qPCR confirmed that ESRRA binds to both motifs (Figure 4I).

DSN1 is a subunit of MIS12 complex, which is highly conserved kinetochore protein and composed of Mis12, Pmf1, Nsl1 and Dsn1. It has been revealed that MIS12 complex is indispensable in mitosis, which can interact with Ndc80 and Knl1 complexes, ultimately forming KMN network and bring microtubule binding activities together at the right time and place. In this process, DSN1 will be activated by Aurora B thus causing MIS12 to bind centromere protein at centromeres 12. Recently, an original research described a noncanonical function of Mis12 in regulating mouse oocytes' meiotic G2M transition, in which researchers found that Mis12 depletion hinders germinal vesicle breakdown by reducing cyclin B1 12. Based on the results of analyses we have obtained and findings proved by other scholars, we continued exploring if ESRRA would bring impact on cell cycle and via which pathway it exerts influences.

### Downregulating ESRRA suppresses cell-cycle G2M transition in GC cells via DSN1 and CDC25c/CDK1/CyclinB1 pathway

**Figure 5:**
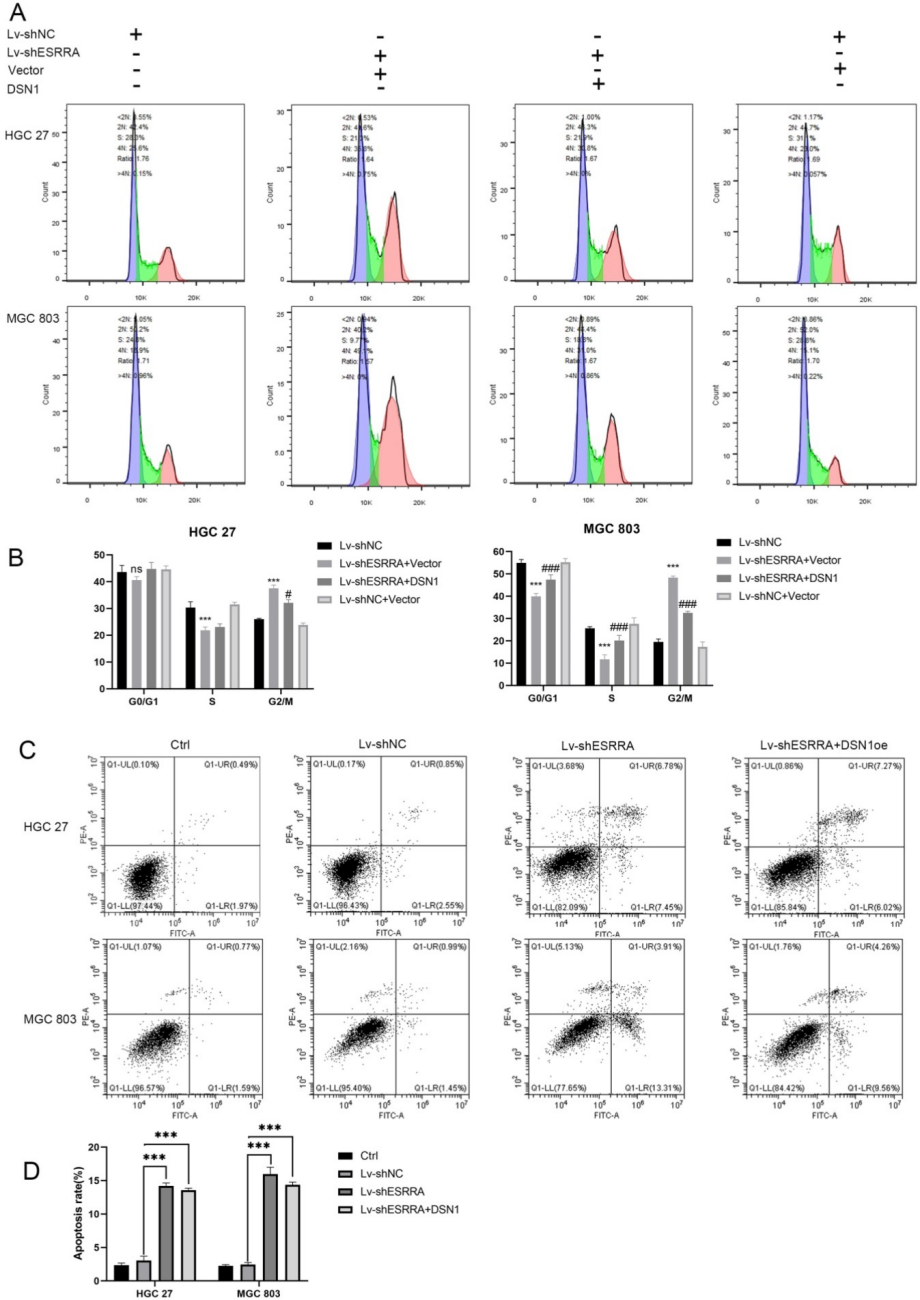
** ESRRA depletion induced apoptosis and G2M arrest.** A, B. The cell cycle distribution was analyzed by flow cytometry and bar charts showed below depicted the percentages of cell cycle distribution. G2M arrest was presented when ESRRA was downregulated. However, when we overexpress DSN1 at this basis G2M arrest was rescued. C, D. Apoptosis assay was also performed by flow cytometry, the apoptosis rates in both ESRRA silencing cell lines were increased but overexpress DSN1 at this basis failed to rescue the apoptosis status. Significant differences are indicated by *P < 0.05, **P < 0.01 and ***P < 0.001 when comparing Lv-shESRRA+Vector with negative control (Lv-shNC); ^#^P < 0.05,^ ##^P < 0.01 and^ ###^P < 0.001 when comparing Lv-shESRRA+DSN1 with Lv-shNC.

**Figure 6:**
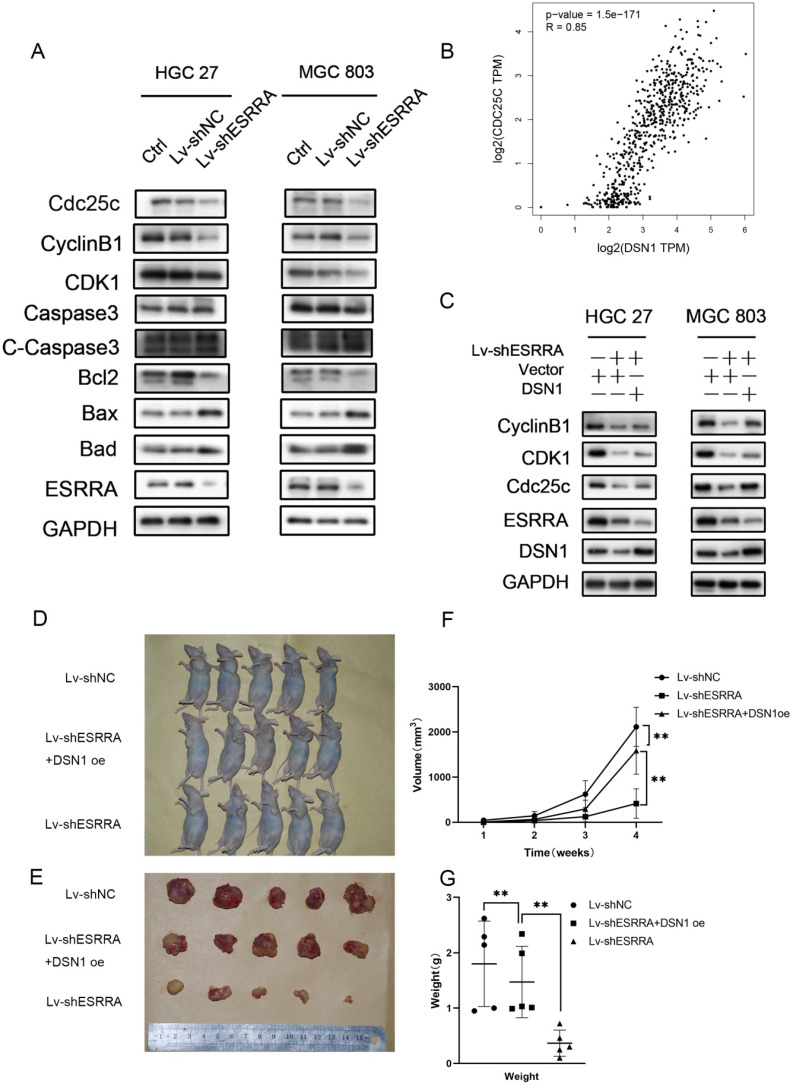
** Silencing ESRRA suppresses cell-cycle G2M transition in GC cells via DSN1 and CDC25c/CDK1/CyclinB1 pathway and suppressed tumorigenesis of gastric cancer GC *in vivo*.** A, C. Western blot analysis of CDC25c, CDK1, CyclinB1 in ESRRA silencing cells and ESRRA silencing + DSN1 overexpression cells. The mentioned protein expression levels were rescued by DSN1 overexpression. However, C-Caspase 3, Bcl2, Bax and Bad expressions were only decreased when ESRRA was downregulated and were not rescued by DSN1 upregulating. (Negative results in this part were not shown in our paper). B, Correlation Analysis between DSN1 and CDC25C was compute via Gepia2 with TCGA tumor, TCGA Normal and GTEx database. (Spearman coefficient was used in figure and when Pearson coefficient was used R=0.69, P=0.). D. MGC 803 control, MGC 803 Lv-shESRRA+DSN1 overexpression as well as MGC 803 Lv-shESRRA cells (5 x 10^6^ in 200 µL PBS) were injected into the left axilla of mice according to groups and animals were continued to feed for 4weeks. E. Subcutaneous tumors excised from mice were shown. F, G. tumor volumes and tumor weights in each group were shown. The tumor volumes were measured by caliper and estimated (0.5 × width^2^ × length) weekly for 4 weeks. Significant differences are indicated by *P < 0.05, **P < 0.01 and ***P < 0.001.

Flow cytometry analysis was performed to explored the underlying mechanism in GC progression. Results showed that cell cycle was strikingly arrested at the G2M phase in the ESRRA depleted cells (Figure 5A, 5B). Thus, we focused on detecting G2M checkpoint related protein markers with western-blot, including CDC25C, Cyclin B1 and CDK1. As seen in Figure 6A, all protein mentioned were downregulated in ESRRA-silencing GC cells when comparing with control and Lv-shNC cell lines. To sum up, ESRRA have great impact on GC cell cycle regulation and ESRRA silenced GC cell would be arrested at G2M by downregulating Cyclin B1 and CDK1.

Cyclin B1 is a pivotal cyclin, which is involved in G2M phase transition and essential for mitosis initiation 13. It has revealed cyclin B1 plays a facilitating role in various malignant tumor development 14, such as breast, colorectal, lung cancer and so on. Besides, upregulated cyclin B1 in solid tumors are also related to poorer prognoses including but not limited to breast cancer, gastric cancer, and esophagus carcinoma.

It has been elucidated that CDC25A and CDC25B activate cyclin B1/CDK1 complex. However, CDC25C plays the key role maintaining complete cyclin B1/CDK1 complex activation and determines G2M checkpoint. Activated CDC25C is required on dephosphorating CDK1 Thr14 and Tyr14 residues. After dephospholation, cyclin B1/CDK1 complex enters nuclear and initiates cell division 15. However, from the results of RNA-seq assay we noticed that CDC25C was downregulated in siESRRA group comparing with NC group of both HGC 27 and MGC 803 cell lines. We wondered whether ESRRA regulate CDC25C-CDK1-Cyclin B1 pathway directly or via DSN1, so correlation between DSN1 and CDC25C was compute via GEPIA2 (Figure 6B) and western-blot assays were conducted and finally confirmed DSN1 functions as the bridge of ESRRA and CDC25C-CDK1-Cyclin B1 pathway (Figure 6C).

### ESRRA silencing promotes apoptosis in GC cells

Flow cytometry analysis was also used to detect the impacts on ESRRA depleting GC. As seen in Figure 5C, the proportions of apoptotic cells in ESRRA-silencing HGC 27 and MGC 803 were higher than negative control and Lv-shNC group. However, the apoptosis rate in ESRRA-silencing+DSN1 overexpression group was not redeemed. We also further evaluated the expression levels of apoptosis makers such as cleaved caspase-3, BCL-2, Bad and Bax. As shown in Figure 6A and Figure S1E, the expression level of cleaved caspase-3, Bad and Bax were higher in ESRRA-silencing groups. Besides, the tendency of BCL-2 showed opposite way. Taken together, our results showed that downregulating ESRRA favored GC apoptosis but the apoptosis can't be rescued by DSN1 overexpression.

### ESRRA silencing suppressed GC growth *in vivo*

In order to investigate the function of ESRRA and DSN1 in GC growth *in vivo*, subcutaneous xenograft nude mice models were also constructed with MGC 803 Lv-shNC, MGC 803/Lv-shESRRA and MGC 803/Lv-shESRRA+DSN1 overexpression. As shown in Figure 6D-G, MGC 803/Lv-shESRRA had impaired ability in tumor formation when comparing with MGC 803 Lv-shNC, but MGC 803/Lv-shESRRA+DSN1 rescued to a degree (Figure 6F, G). After the s.c tumors were harvested, we performed IHC with Ki-67 to detect the proliferation status among these three groups. MGC 803 Lv-shNC group showed strong level of staining, MGC 803/Lv-shESRRA+DSN1 overexpression showed moderate level and MGC 803/Lv-shESRRA was weakly stained (Figure S3D). To sum up, the staining results indicated that ESRRA is of great importance in GC cells' tumorigenesis and tumor growth. Cell proliferation ability would be impaired when ESRRA was silenced and can be redeemed to a degree when DSN1 overexpressed at the same time.

## Discussion

Our data indicated that comparing to normal gastric mucosa tissue and normal gastric epithelial cell lines ESRRA was predominantly upregulated in clinical GC biopsies and GC cell lines. In immunohistochemistry assays, the association between higher ESRRA expression and poorer survival prognoses covering 246 patients had been discovered, which is in accordance with Kaplan-Meier Plotter online analysis results. Based on all the findings above, we hypothesized that ESRRA overexpression in gastric cancer favors GC growth and viability, supports tumor cell invasion and metastatic abilities.

Afterwards, we conducted CCK8 assays, colony formation assay, Transwell migration and Matrigel cell invasion assays. All the results above showed that ESRRA silencing suppressed GC cell viability, proliferation, invasive and migrative abilities. Flow cytometry analysis focusing on cell cycle and apoptosis manifested that G2M arrest and increased apoptosis ratio were characteristics in ESRRA silencing group.

Considering ESRRA plays the role of transcription factor which doesn't respond to steroid hormones but takes effects by binding EREs and ERREs. We turned to RNA-seq to explore the possible gene regulated by ESRRA and validate it by ChIP-DNA electrophoresis and ChIP-qPCR. Results supported that ESRRA regulates DSN1 by binding EREs within its promoter region, which was in accordance with results obtained from Harmonizome (CHEA Transcription Factor Targets dataset and ENCODE Transcription Factor Binding Site Profiles dataset).

DSN1, a conserved component of kinetochore protein complex which known as MIS12, is indispensable for proper kinetochore assembly and chromosome-spindle attachment in mitosis process. It also reported that DSN1 depletion would lead to chromosome misalignment 16 as well as mitotic delay 17. Studies have revealed that DSN1 high expression is related to poor survival in patients with hepatocellular carcinoma and colorectal cancer progression 18, 19. Spindle assembly involves multiple rounds of microtubule attachment and detachment on kinetochores. When individual chromosomes fail to attach properly, mitotic phase would be inevitably delayed 20, 21. As a result, we focused on the effects that DSN1 took in cell cycle regulation. Similarly, in the research focusing on DSN1 expression and colorectal cancer progression, evidences also showed that down-regulating of DSN1 favor cyclin B1 expression and increase G2M arrest, which is in accordance with the research conclusion drawn above. What's more, deregulated DSN1 may contribute to uncontrolled cell cycle and cancer development.

Recently, Feng et al. 22 utilized integrated analysis of array-based comparative genomic hybridization (aCGH) and gene expression values to identify significant differentially expressed genes (DEGs) with copy number changes that correlated with GC progression and pathophysiology. In significant modules, DSN1 was spotted as upregulated gene with amplification, which is associated with cell cycle. We also explored the DSN1 expression status based on sample types, individual stages, and tumor grade in gastric cancer with UALCAN (http://ualcan.path.uab.edu/index.html). As shown in Figure S3A-S3C, the expression of DSN1 is obviously higher in primary tumor with various stages and grades than normal tissues.

Our results showed that ESRRA regulate DSN1 expression by binding to its EREs located on promoter region. When ESRRA was knocked-down, the expression level of DSN1 was reduced accordingly (Figure 6C). In cell cycle analysis, G2M arrest brought by ESRRA downregulation would be rescue by DSN1 overexpression but apoptosis inducing was not rescued. Studies mentioned above have shown DSN1 exert impact on cell cycle by regulating cyclin B1 expression directly, we further focused on CDC25C/CDK1/CyclinB1 signaling pathway and conducted western-blot, finally found that ESRRA silencing hindered CDC25C/CKD1/CyclinB1 pathway and cause G2M arrest, which could be rescued by DSN1 overexpression. In the experimental animal models, we constructed with MGC 803 control, MGC 803 Lv-shESRRA+DSN1 overexpression group as well as MGC 803 Lv-shESRRA group. Not only mean tumor volumes and weights in these 3 groups were from largest to smallest accordingly, but also ki-67 staining was presented from heaviest to weakest.

G1/S and G2/M checkpoints are both critical for damaged DNA double strands to get repaired. If the repairation fails, cells would inevitably progress to apoptosis. In our experiment, we testified that apoptosis rate was higher in ESRRA silenced group than control group by flow cytometry and western-blot. However, HGC 27 and MGC 803 cell apoptosis tendency caused by ESRRA downregulation was not rescued by DSN1 upregulation. These results were quite different from the phenomena we observed in cell cycle assays but in accordance with the conclusion drawn from a study focusing on colorectal cancer. Chuang et al. 19 found that DSN1 knockdown gave rise to G2M arrest without affecting cell growth or apoptosis in colorectal cancer cell line, which suggest colorectal cancer can still survive even there is defect caused by DSN1 depletion. Nevertheless, specific mechanism for cell survival when DSN1 depletion is still unclear and calls for further study.

CDC25C/CDK1/CyclinB1 pathway is critical in G2 checkpoint. CyclinB1 progressively increase though G1 and S phase and reach its peak in G2 phase then form complex with CDK1 23. It has been documented that CDK1 is activated on centrosomes 24, Cyclin B1-CDK1 binds to the microtubules, chromosomes and to unattached kinetochores in the prometaphase 25. Only dephosphorylated CDK1/CyclinB1 complex is active, allowing transition to mitosis 26.

In our study, we detected CDK1 and CyclinB1 level by western-blot and found that comparing with control and Lv-shNC group, their expressions were downregulated in Lv-shESRRA group. When DSN1 was overexpressed, their levels were rescued to a degree. The conclusions we drawn from western-blot assays comply with findings we got from flow cytometry when detecting cell cycle arrest.

Lv et al. 27 found that CyclinB1 inhibition not only induce G2M phase arrest in hepatocellular carcinoma (HCC), but also suppress EMT. They detected the expression of EMT-associated protein vimentin, N-cadherin, and E-cadherin via western-blot. Results showed knockdown CyclinB1 in HCC cell lines restrained expression of vimentin and N-cadherin while enhancing E-cadherin expression. We also observed that ESRRA silencing suppressed EMT transition and we speculate that ESRRA-DSN1-CDC25C/CDK1/CyclinB1 may explain this phenomenon.

## Conclusions

In summary, we observed that ESRRA was highly expressed in GC tissues and cell lines. ESRRA silencing impaired proliferation, viability, migration, and invasion of GC cell. By RNA-seq, dual-luciferase assays, and ChIP assays later analyses, we confirmed ESRRA regulate DSN1 as transcription factor by binding its promotor region. Subsequently, we verified that ESRRA downregulation induced G2M cycle arrest by suppressing CDC25C/CDK1/CyclinB1 pathway via regulating the expression of DSN1 by flow cytometry and western-blot. Collectively, our data provide solid evidence that ESRRA play key role in GC progression and development, which suggests that drug targeting ESRRA could be used as a potential therapeutic method for gastric cancer.

## Supplementary Material

Supplementary figures and tables.

## Figures and Tables

**Table 1:** Association of ESRRA expression with GC clinicopathological characteristics

	Total cases	ESRRA expression		
Clinic Characteristics	N=246	Low(n=83)	High(n=163)	P value
Sex				
Male	124	47	77	**0.179(NS)**
Female	122	36	86	
Age				
≤60	80	30	50	**0.392(NS)**
>60	166	53	113	
Differentiation Status				
Well or Moderate	142	42	100	**0.133(NS)**
Poor	104	41	63	
T classification				
T1+T2	86	50	36	<0.001
T3+T4	160	33	127	
Lymph node metastasis				
N0	102	47	55	0.006
N1-3	144	36	108	
Distant metastasis				
M0	200	77	123	0.009
M1	46	6	40	
TNM stage				
I+II	153	63	90	0.002
III+IV	93	20	73	

Tumor TNM stage was based on American Joint Committee on Cancer. P<0.05 was recognized as statistically significant.
