# Supplementary material for: ESRRA promotes gastric cancer development by regulating the CDC25C/CDK1/CyclinB1 pathway via DSN1
Source: Int J Biol Sci. 2021 May 10;17(8):1909–24. doi: 10.7150/ijbs.57623 (PMC8193261; doi:10.7150/ijbs.57623)
Supplement: Supplementary file 1 — Supplementary figures and tables. [file ijbsv17p1909s1.pdf]

Supplementary Materials

pGL3-DSN1-WT-luc containing the -2000 bp to -1 bp promoter sequence of DSN1. DSN1 promoter region's -2000bp to -1bp sequence was obtained from http://genome.ucsc.edu and the highlighted part are binding sites.

SEQUENCE:

tcaagtaacagggattataggtgcataccaccacacctggctaattttgtatttttagtagagacagggttttgctatgttggccag
gctggtctcaaactcctgacctcaactgatccgcaggcctcagcctcccaaactgctgggattagaggcgtgagccaccacaccc
agcctaatgacacagtgccttataggggaaaaaaatgtgtgcctcctgtggacaggagggagccatgcaacaattcaaagcag
ggaaacgatgtagaagatgtattgggtctgtattccatgaaatcattttctcattgtgccccaggatggccagccctagctgggaa
ggaacactgagttctcttcctggcacgttgacagagaggtaaaaggaactgggtacctctcaaccccagaaagtgatattcagg
gagccaagggcaaatctctgctttcagaatggctgctgcagccagcaggctggaaccacagggtggaaatttcaggaaggcag
atttttgtctcccaccagaaaacttttccaaggaccacaactgctatggaaggatgaccacggtcagctccagctgcctctctccaa
cccctcttttggactgtgggctgcttataattcaatatttggaagtttttctgtgaaagaaaggactgtgcacatccaaggggacatt
ttggtggcgtcgtgctgggtgctgaggaagcagcccgctctctggagcctcctagaccagcatgggatagtggagcaggaaca
gattgggctcctgggagcacagaaaggacagttggttttcagcctaaggccagagagaatgcttggagagaaatcaatcattcc
ttagctgaggcttaaaattcgaggatgtggccgggcgcagtggctcatgcctgtaatcccagctctttgggaggccgaagtgggt
ggatcacttgaggtcaggagttcgcgaccagcctggccaacattttagtggccgtctctactaaaaatacaaaaattagccaggc
gtggtggtgggcgcctgtaatcccagctactcgggaggctgaggcaggagaatcgcttgaacccgaacccgggaggcggag
gttgccgggagccgagatcgcgccactgcactctagcctgggcgacagagcgagactccgtctcaaaatacaaaaaaattgga
ggatgtagctaggaaaagcagggaaagccttcccaggcagagggaacagcgcctgggaatatagcgcctcaaagggcatct
gatgccctgtccccgtcctacccctaaggaccagcccgacctgccgcacccccgaccccgactaaggacactgcgaccacttgg
gggcgctgcgacctcctcccgggtgaaatgaaacccgagggcaggcccccacccaccaagcaagtaaagcaagccgtttgag
ggagtggcctggaaatgagagagaaaaagtcctagagcaggagtgggatagactgtgaggcaaactgagctcagagctcca
gtccaccctggggagagtcacggtccacattttgggagttaaggagataaagggagtgggaaccagagcctagcgacgggg
acagggcatcagatgccctttgaggcgctgtattcctccggcaagaatctctccgggcctctgtttcccccatcttagatgggctctg
gggccgtcgggcgcaaaaggacacgatagtgtggcgggtcccggaaagggactcccgggcggggtgttgcggggaggtg
gtgacgtggccagacaggacctaaaggacttggactagaggagcggagctggcaggagcgttggacggcggtgtacgcatg
cgcgtcgggcggcggtgtacgcatgtgcatcgccggaggcaggcgttctgcgcgcgcgtgcccgagaaactgacggccgcgc
atgcgcccttggttggcgggagtttcggaggcggtgaccgtgacgtagaaggt

Primer details are as following:

site 1 forward, 5′- AGTTTAAGAGGCAGGCAAGCA-3′

site 1 reverse, 5′- GCAGTTGTGGTCCTTGGAAAA-3′;

site 2 forward, 5′- TTGGACTGTGGGCTGCTTAT-3′

site 2 reverse, 5′- CCCAGGAGCCCAATCTGTTC-3′.

Mutant SEQUENCE:

tcaagtaacagggattataggtgcataccaccacacctggctaattttgtatttttagtagagacagggttttgctatgttggccag
gctggtctcaaactcctgacctcaactgatccgcaggcctcagcctcccaaactgctgggattagaggcgtgagccaccacaccc
agcctaatgacacagtgccttataggggaaaaaaatgtgtgcctcctgtggacaggagggagccatgcaacaattcaaagcag
ggaaacgatgtagaagatgtattgggtctgtattccatgaaatcattttctcattgtgccccaggatggccagccctagctgggaa
ggaacactgagttctcttcctggcacgttgacagagaggtaaaaggaactgggtacctctcaaccccagaaagtgatattcagg
gagggttcccgttttctctgctttcagaatggctgctgcagccagcaggctggaaccacagggtggaaatttcaggaaggcagat
tttgtctcccaccagaaaacttttccaaggaccacaactgctatggaaggatgaccacggtcagctccagctgcctctctccaacc
cctcttttggactgtgggctgcttataattcaatatttggaagtttttctgtgaaagaaaggactgtgcacatccaaggggacatttt
ggtggcgtcgtgctgggtgctgaggaagcagcccgctctctggagcctcctagaccagcatgggatagtggagcaggaacag

attgggctcctgggagcacagaaaggacagttggttttcagcctaaggccagagagaatgcttggagagaaatcaatcattcctt
agctgaggcttaaaattcgaggatgtggccgggcgcagtggctcatgcctgtaatcccagctctttgggaggccgaagtgggtg
gatcacttgaggtcaggagttcgcgaccagcctggccaacatttttagtggccgtctctactaaaaatacaaaaattagccaggcg
tggtggtgggcgcctgtaatcccagctactcgggaggctgaggcaggagaatcgcttgaacccgaacccgggaggcggaggt
tgccgggagccgagatcgcgccactgcactctagcctgggcgacagagcgagactccgtctcaaaatacaaaaaaattggagg
atgtagctaggaaaagcagggaaagccttcccaggcagagggaacagcgcctgggaatatagcgcctgtttaaagtagtgat
gccctgtccccgtcctacccctaaggaccagcccgacctgccgcacccccgaccccgactaaggacactgcgaccacttgggg
gcgctgcgacctcctcccgggtgaaatgaaacccgagggcaggcccccacccaccaagcaagtaaagcaagccgtttgaggg
agtggcctggaaatgagagagaaaaagtcctagagcaggagtgggatagactgtgaggcaaactgagctcagagctccagtc
caccctggggagagtcacggtccacattttgggagttaaggagataaagggagtgggaaccagagcctagcgacggggaca
gggcatcagatgccctttgaggcgctgtattcctccggcaagaatctctccgggcctctgtttcccccatcttagatgggctctggg
gccgtcgggcgcaaaaggacacgatagtgtggcgggtcccggaaagggactcccgggcggggtgttgcggggaggtggtg
acgtggccagacaggacctaaaggacttggactagaggagcggagctggcaggagcgttggacggcggtgtacgcatgcgc
gtcgggcggcggtgtacgcatgtgcatcgccggaggcaggcgttctgcgcgcgcgtgcccgagaaactgacggccgcgcatg
cgcccttggttggcgggagtttcggaggcggtgaccgtgacgtagaaggt

Supplementary figure 1

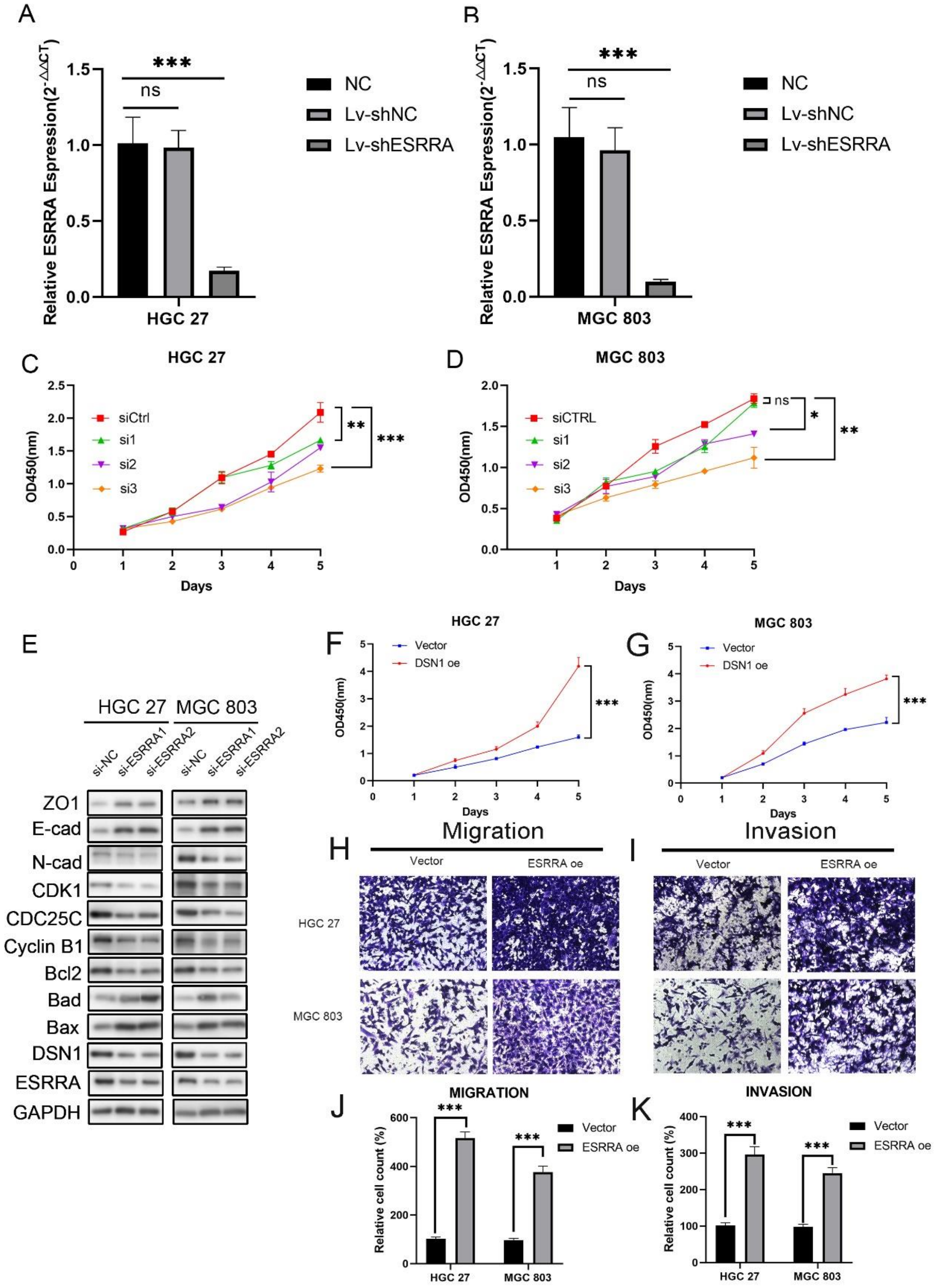

Verification of knock-down efficacy of Lv-shESRRA and cell viability reduction caused by siRNA. Supplementary Figure 1. A, B. The efficacy of Lv-shESRRA for knock-down in HGC 27 and MGC803 was determined via qRT-PCR. C, D. HGC 27 and MGC 803 GC cells viability was decreased when ESRRA was silenced by siRNA and si3 showed greater potency among 3 siRNA sequences. E. Western-blot focusing on key

proteins affected by ESRRA silencing was conducted to avoid off-target effect, sequences of si2 and si3 were used in this part. F, G. ESRRA overexpression favored HGC 27 and MGC 803 proliferation abilities. H, I, J, K. Cells' migrative and invasive capacities were also elevated then ESRRA was upregulated.

Supplementary figure 2

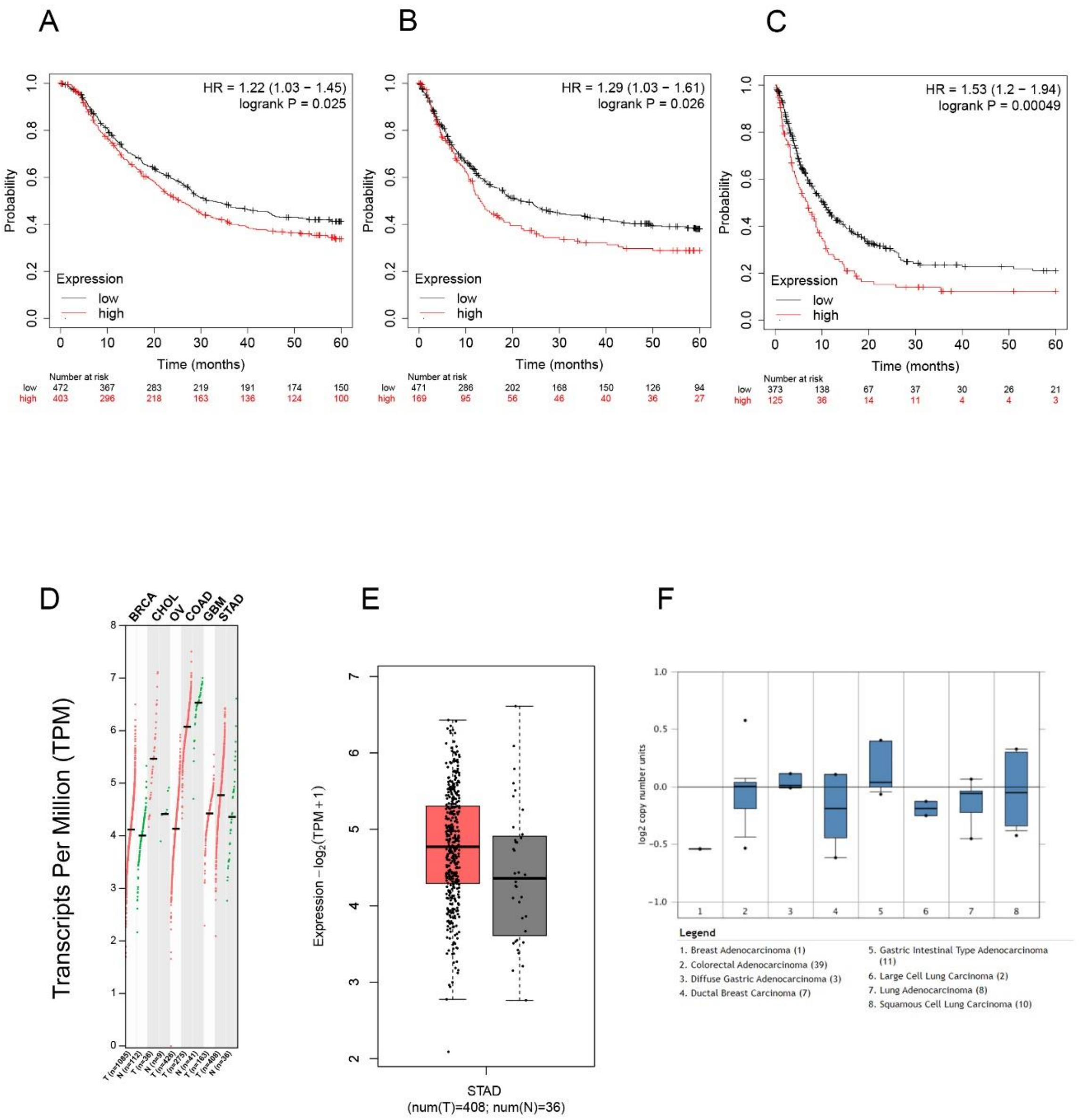

ESRRA is upregulated in GC and higher expression is related to poorer prognosis. A, B, C. High expression of ESRRA in GC patients tend to have poor prognosis. A. The graph focused on overall survival (OS), which is based on Kaplan-Meier plotter Gastric

Cancer database and follow up threshold is 60 months. Low expression cohort median survival is 31.33 months and high expression cohort median survival is 25.2 months. B. The graph focused on first progression (PF), which is based on Kaplan-Meier plotter Gastric Cancer database and follow up threshold is 60 months. Low expression cohort median survival is 21.73 months and high expression cohort median survival is 13.1 months. C. The graph focused on post progression survival (PPS), which is based on Kaplan-Meier plotter Gastric Cancer database and follow up threshold is 60 months. Low expression cohort median survival is 10.3 months and high expression cohort median survival is 7 months. To sum up, GC patients with higher ESRRA expression tend to have shorter survival period, get recurrence sooner and progress faster after recurrence. D, E, F. ESRRA expression is increased in GC tissues. D. ESRRA expression level in GC and other tumor types in which have been explored and published so far. ESRRA level in GC is second to cholangiocarcinoma and ranks third among them. E. Mean expression level of ESRRA in GC tissue is higher than that in normal tissue (data is extracted from GEPIA2 when matching TCGA normal data). F. Jaiswal Multi-cancer database showed ESRRA is upregulated in gastric intestinal type adenocarcinoma as well as diffuse gastric adenocarcinoma.

Supplementary figure 3

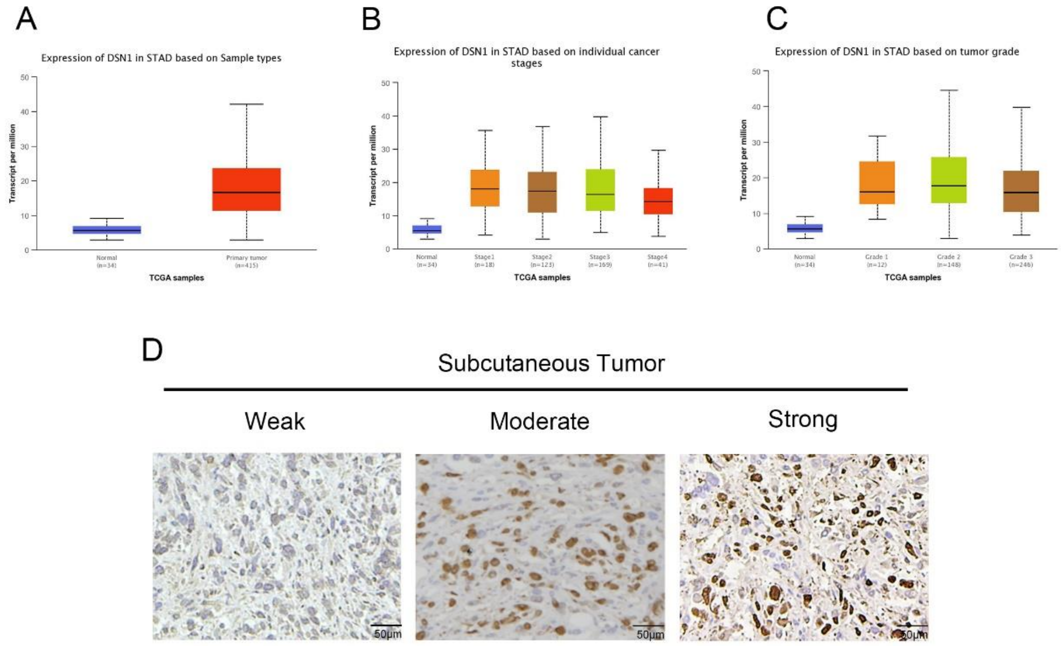

A, B, C. DSN1 is upregulated in GC and tumor in higher grades as well as later stages showed higher expressions. D. Ki-67 staining intensity in MGC 803/Lv-shESRRA group,

MGC 803/Lv-shESRRA+DSN1 overexpression group and MGC 803 Lv-shNC group. They were stained from weak to strong accordingly.
